# Routine Pathology and Postoperative Follow-Up are Not Cost-Effective in Cholecystectomy for Benign Gallbladder Disease

**DOI:** 10.1007/s00268-018-4619-5

**Published:** 2018-04-25

**Authors:** Pim B. Olthof, Madelon J. H. Metman, Ronald R. de Krijger, Joris J. Scheepers, Daphne Roos, Jan Willem T. Dekker

**Affiliations:** 1 Department of Surgery, Reinier de Graaf Gasthuis, Reinier de Graafweg 5, 2625 AD Delft, The Netherlands; 2 Department of Experimental Surgery, Academic Medical Center, University of Amsterdam, Amsterdam, The Netherlands; 3 Department of Pathology, Reinier de Graaf Gasthuis, Delft, The Netherlands

## Abstract

**Introduction:**

The incidence of gallstone disease is increasing and represents a strain on healthcare systems worldwide. Following cholecystectomy, gallbladder specimens are generally submitted for histopathologic examination and the diagnostic yield of this strategy remains questionable. This study aimed to evaluate the usefulness of routine pathologic examination of the gallbladder specimens and investigate the results of routine postoperative follow-up visits.

**Methods:**

All cholecystectomies performed between January 2011 and July 2017 at a single center were evaluated. All gallbladder specimens were routinely pathologically examined. The outcome parameters were the macro- and microscopic gallbladder anomalies at pathology and the reported symptoms during routine follow-up visits 2–6 weeks after surgery.

**Results:**

In the study period a total of 2763 patients underwent cholecystectomy, of which 2615 had a postoperative visit in the outpatient clinic. Seventy-three patients (3%) complained of persistent abdominal pain, and 29 of these patients were referred for further treatment, resulting in a resolution of symptoms in 97%. Of all gallbladder specimens, 199 (7%) displayed macroscopic anomalies and in four (2%) of these, gallbladder carcinoma was diagnosed.

**Discussion:**

Selective pathologic examination of gallbladder specimens in case of macroscopic anomalies appears justified. Also routine follow-up after cholecystectomy appears not useful since 97% of patients do not report any symptoms at follow-up. A selective pathology and follow-up strategy could save significant healthcare costs.

## Introduction

Gallstone disease accounts for numerous hospital visits and high healthcare costs in the Western world. The prevalence of gallstones in adults is around 10–20% and increasing in line with rising obesity rates and the aging population [1–3]. Approximately one in four patients with gallstones undergoes cholecystectomy over a 10-year follow-up period. These statistics make gallstone disease the second most expensive disease entity in the USA with an estimated cost of $6.5 billion annually [4].

Traditionally, all gallbladder specimens removed during cholecystectomy are sent for histopathologic examination [5]. The possibility of an incidental diagnosis of gallbladder carcinoma is the primary rationale for this pathologic evaluation; however, reported incidences are usually well below 1% [6]. More recently, some studies have questioned the relevance and effectiveness of this routine examination [7–9]. Although gallbladder carcinoma itself has a poor prognosis [10], the impact of an incidental diagnosis is most often inconsequential since cholecystectomy alone usually suffices [11]. Since the incidence of gallstones and cholecystectomy is rising [10], the relevance and cost of this routine gallbladder specimen pathologic evaluation is of increasing relevance. Also, cholecystectomy usually results in a complete resolution of symptoms in the majority of cases [12], which questions the relevance of the routine follow-up visits that are usually scheduled if gallbladder specimens are sent for routine pathology. Evidence-based medicine and cost-effective treatment are essential and relevant in order to keep health care affordable [13].

The aim of this study was to evaluate the usefulness of routine pathologic examination of the gallbladder following cholecystectomy and investigate the outcome of routine postoperative follow-up visits. In addition, the yearly number of gallbladder specimens submitted for histopathologic examination was compared to the number of cholecystectomies in the Netherlands.

## Methods

All consecutive patients who underwent elective or emergency cholecystectomy between January 2011 and June 2017 in a single major teaching hospital in the Netherlands were included. Cholecystectomies combined with other procedures were excluded since the cholecystectomy was not the primary indication for surgery with the exception of cholecystectomies combined with umbilical hernia repair. All consecutive cholecystectomy procedures were identified from the operating room digital planning system. All clinical data were obtained from the electronic medical records. Potential visits to general practitioners or other hospitals were not available for data analyses.

The indications for cholecystectomy were uncomplicated symptomatic gallstone disease and complicated gallstone disease, as acute cholecystitis, (acute) biliary pancreatitis, previous endoscopic retrograde cholangiopancreatography (ERCP), and gallbladder polyps of at least 1 cm, multiple or growing polyps. Uncomplicated symptomatic gallstone disease was defined in most cases by a typical patient history supported by ultrasound confirmation of the presence of gallstones [14]. Patients with acute cholecystitis were considered for cholecystectomy according to national guidelines [15] within 1 week of the onset of symptoms or for delayed surgery after 6 weeks. If required, percutaneous gallbladder drainage was performed as a bridge to surgery, or in patients considered unfit for surgery at that time. In patients with acute biliary pancreatitis, cholecystectomy was usually performed after 6 weeks, and in selected patients with mild pancreatitis directly after discharge from the hospital.

Patients with an uncomplicated procedure and postoperative course were usually discharged at postoperative day one. Selected patients were discharged the same day. Patients with a perforated or gangrenous cholecystitis were treated postoperatively with one or more days of intravenous antibiotics. A single follow-up visit at the outpatient clinical was scheduled to evaluate the clinical course and discuss the pathology results. The visit was planned 2–3 weeks after surgery, which was altered to around 6 weeks postoperatively since 2014.

### Outcome variables

All medical records were reviewed for postoperative complications that occurred within 30 days after surgery complications were graded according to Dindo et al. [16]. Bile duct injury was classified as (A) cystic duct, or aberrant or peripheral hepatic radicles leaks, (B) major bile duct leaks, (C) bile duct strictures without leakage, or (D) complete transection of the bile duct [17].

At the postoperative outpatient visits, any reported post-cholecystectomy diarrhea, or abdominal complaints and the treatment were scored. In addition, any surgical site infection diagnosed at the outpatient visit was scored.

All gallbladder specimens were analyzed according to a standard grossing protocol at the Department of Pathology. The presence of gallstones and polyps, wall thickness, and any other gross abnormalities were noted. Typically, a single block was taken, in which a cross section of the neck of the gallbladder and a representative part of the wall were included for microscopy. The presence of gallstones, inflammation, dysplasia, and malignancy were scored in the current database.

### National data

The annual total number of gallbladder specimens submitted for pathologic examination between 1991 and 2016 in the Netherlands was obtained from the online data repository PALGA, the nationwide network and registry of histo- and cytopathology in the Netherlands [18]. The total number of cholecystectomies per year in the Netherlands was obtained from the online data repository from the central office of statistics of the Netherlands from 1995 to 2010. Since the data after 2010 were not available yet, the total number of cholecystectomies in 2015 was estimated from the healthcare insurance database, based on the number of reimbursed cholecystectomy procedures that year using data of the Dutch Healthcare Authority.

## Results

Between January 2011 and July 2017 a total of 2763 consecutive patients underwent cholecystectomy. The majority of patients were women (69%) with uncomplicated symptomatic gallstone disease (49%) who underwent laparoscopic cholecystectomy (49%). Baseline patient and disease characteristics are displayed in Table 1. The outcomes and complications are displayed in Table 2. The major complication rate and reoperation rate were 3 and 1%, respectively. The most frequent complications were surgical site infections. Most patients were discharged the day after surgery (75%).

**Table 1:** Patient and disease characteristics

	*N* = 2763
Age, years, median (IQR)	51 (39–62)
Female gender, *n* (%)	1920 (69)
Body mass index, median (IQR)	27.3 (24.5–30.7)
ASA classification, *n* (%)	
I	1232 (45)
II	1333 (48)
III	195 (7)
IV	3 (0)
Indication for surgery, *n* (%)	
Symptomatic gallstones	1784 (65)
Cholecystitis	508 (18)
Biliary pancreatitis	114 (4)
Following ERCP	265 (10)
Gallbladder polyps	78 (3)
Gallbladder anomaly on imaging	14 (1)
Previous abdominal surgery, *n* (%)	921 (33)
Laparoscopic approach, *n* (%)	2750 (99.5)
Conversion rate, *n* (%)	73 (3)

**Table 2:** Outcomes

	*N* = 2763
Morbidity, any*, n* (%)	252 (9)
Morbidity, Dindo IIIa or higher, *n* (%)	95 (3)
Morbidity, highest Dindo grade, *n* (%)	
I	108 (4)
II	49 (2)
IIIa	37 (1)
IIIb	47 (2)
IVa/b	10 (0.36)
V	1 (0.04)
Morbidity, specified, *n* (%)	
Surgical site infection	76 (3)
Readmission	55 (2)
Biliary leakage	39 (1)
Intra-abdominal abscess	19 (1)
Urinary complication	12
Pneumonia	11
Electrolyte disturbances	7
Ileus	3
Pulmonary embolism	3
Sepsis	1
Pancreatitis	3
Phlebitis	6
Infected hematoma	8
Delirium	3
Cardiac complications	4
Bleeding	10
Lost intra-abdominal gallstone	1
Incisional hernia	5
Bowel perforation	2
Other	12
Reoperation rate, *n* (%)	17 (1)
Postoperative hospital stay, days, median (IQR)	1 (1–1)

Of all patients, 2615 (95%) were subjected to at least one follow-up visit in the outpatient clinic (Table 3). At these outpatient visits, 32 patients complained of diarrhea after cholecystectomy, which did not require any treatment or follow-up in any of these patients since it is considered normal after cholecystectomy [19]. At these follow-up visits, 33 patients were diagnosed with a surgical site infection, either ongoing or resolved without any hospital treatment. Of all 2615 patients, 73 (3%) complained of persistent abdominal complaints, resulting in a complete resolution of symptoms in 97% of patients. Of these patients with abdominal complaints, 29 were referred to another specialty for further treatment, 25 were subjected to further diagnostic tests or treatment with antacids, and 19 patients did not require any additional treatment or diagnostics. Patients with persistent abdominal complaints had experienced complications more often (19 vs. 9%, *P* < 0.01).

**Table 3:** Follow-up visits and histopathology results

	*N* = 2763
Postoperative follow-up visit, *n* (%)	
Outpatient visit	2589 (94)
Outpatient telephonic consult	27 (1)
Complaints reported at follow-up, *n* (%)	
Diarrhea	32 (1)
Surgical site infection	33 (1)
Abdominal pain	73 (3)
Macroscopic gallbladder anomaly, *n* (%)	199 (7)
Macroscopic finding, *n* (%)	
Focal lesion or thickening	26 (1)
Cystic lesion	15 (1)
Fibrotic	16 (1)
Fragmented	16 (1)
Irregular aspect	16 (1)
Diffuse thickening	110 (4)
Gall stones at pathologic examination, *n* (%)	2168 (78)
Dysplasia, *n* (%)	7 (0)
Malignancy, *n* (%)	4 (0)

In all 2763 patients, the gallbladder specimen was subjected to pathologic examination (Table 3). Macroscopic anomalies were seen in 199 specimens at macroscopic evaluation by the pathologist, including 4 cases of gallbladder cancer, which all exhibited a macroscopic abnormal aspect. None of the macroscopically normal gallbladder specimens harbored malignancy.

The first patient with gallbladder cancer presented with cholecystitis, and the gallbladder wall showed irregularities at macroscopic examination. Pathology showed a T2 tumor, which turned out to be a metastasis from a T3N1 colon carcinoma. The second patient also presented with cholecystitis; the gallbladder was fragmented due to intraoperative rupture. Pathology showed a T2 gallbladder carcinoma. The third patient also presented with cholecystitis, and the specimen had diffuse thickening of the wall with an almost completely compressed gallbladder lumen. A T2 gallbladder carcinoma was diagnosed. The final patient underwent cholecystectomy for uncomplicated symptomatic gallstone disease. Macroscopy showed severe fibrotic changes, and a T2 gallbladder carcinoma was diagnosed.

### No decrease in national gallbladder pathologic examination nationwide

**Fig. 1:**
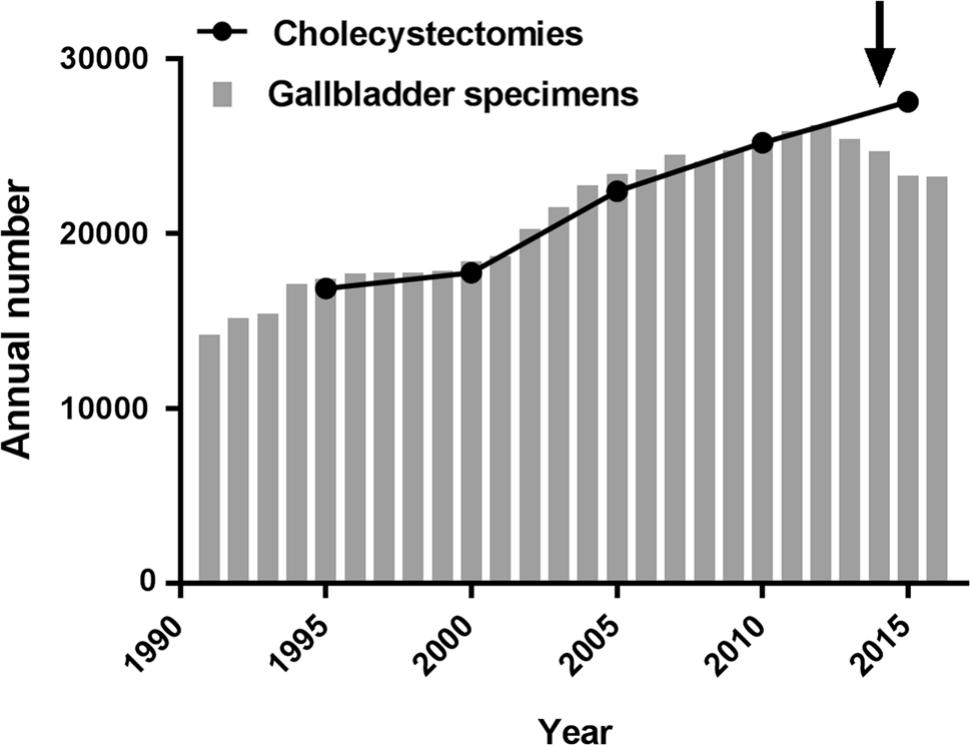
Total number of gallbladder specimens submitted for histopathologic examination annually in the Netherlands (gray bars). Total amount of annual cholecystectomy procedures in the Netherlands (black boxes and connecting line). The arrow indicates the introduction of the revised national guideline with the recommendation to use selective pathology assessment

 Between 1990 and 2012 an increasing number of gallbladder specimens removed for any indication were sent for pathologic examination years nationwide in the Netherlands. A mild decrease was observed from 2013 to 2014 which appears to stabilize around 23,300 annually (Fig. 1). In raisedcontrast, the number of performed cholecystectomies has increased from 16,862 in 1995, to 17,771 in 2000, 22,437 in 2005, and 25,203 in 2010. In 2015, this number further increased to an estimated 27,550 procedures, while the number of specimens sent for pathology declined over the most recent years (Fig. 1).

## Discussion

 In the present study, routine postoperative pathologic examination of 2763 gallbladders resulted in the four diagnoses of gallbladder cancer. Macroscopic anomalies were seen by the pathologist in 199 specimens, and these included the 4 cases of gallbladder cancer. Therefore, submitting only macroscopically abnormal specimens for pathology would reduce the number of specimens by 93% and thereby the number needed to examine from 691 to around 50 in the current study. Routine follow-up visits after cholecystectomy were without consequences, i.e., additional diagnostics, treatment, or referrals in more than 97% of cases.

Some previous studies have reported on the use of routine histology after cholecystectomy. Deng et al. found gallbladder cancer incidence to be 0.32%. Of these 46 patients, gallbladder cancer was already suspected based on preoperative or intraoperative observations. Therefore, the authors concluded selective pathology is justified and more cost-effective [7]. This is, however, an Asian cohort, which might not be comparable to Western cohorts where the incidence of biliary pathology is different [20]. Indeed, Swank et al. reported the incidence of unexpected gallbladder cancer was more frequent in Asia compared to Europe. Again, this review did not support routine pathology. Van Vliet et al. examined 1375 gallbladder specimens and found 8 gallbladder carcinomas all of which were macroscopically abnormal, which would seem to justify selective pathologic examination. Although these reports all suggest selective pathology is justified, they did not focus on postoperative care all together. Usually, follow-up visits are scheduled to discuss the pathology outcomes, and when the specimen is not sent for pathology it remains to be established whether any follow-up visit would be useful.

The current report shows the 2763 specimens sent for histopathologic examination could have been reduced to only 199 specimens. The costs associated with these 2564 unnecessary pathology examinations during the study period are approximately 160,000 euros, according to the Dutch rate of 62.78 euro per specimen. The proposed pathology strategy could save around 25,000 euros annually in our center and 1.6 million euros annually in the Netherlands. Additional costs might be saved by not scheduling standard follow-up visits, only when indicated for pathology follow-up or upon complications or complaints reported by the patient. Most surgical site infections were diagnosed during unscheduled outpatient visits and the reported transient diarrhea did never require treatment, since it is considered normal after cholecystectomy and transient. For 3% of patients with persistent abdominal complaints a follow-up visit could be scheduled upon request. This selective follow-up visits policy could decongest the outpatient clinic and reduce associated costs. Alternatively, patients could be followed up postoperatively with a telephone consult.

 Although the national gallstone disease treatment guideline devised in 2014 does not recommend routine histopathologic examination of gallbladder specimens, the expected 97% decrease in the number of specimens submitted to pathology departments found in this study was not observed nationwide (Fig. 1). A mild decrease in the number of specimens was seen, while the number of cholecystectomies further increased; however, national adherence to the guideline should reduce the gallbladder specimens to a minimal number.

In the current report, several patients with gallbladder polyps were included which is an indication for cholecystectomy in order to obtain a histologic assessment. Interestingly, none of the patients with polyps had gallbladder carcinoma and in the majority of patients no polyps could be identified at either macro- or microscopic assessment. Whether the absence of macroscopic polyps after cholecystectomy of gallbladder polyps is sufficiently accurate to omit microscopic confirmation remains to be established, as the current study is likely underpowered to allow such conclusion for the small group of patients with polyps.

The current study has several limitations. First, the retrospective nature of this study means it could be subject to selection bias despite the fact that all consecutive procedures were included. Second, the number of gallbladder carcinomas was low with 0.1%, which could influence the outcomes and conclusions. However, this report spans the 6.5-year experience of a major teaching hospital and the conclusions are relevant to daily practice in similar centers. Finally, since macroscopically normal gallbladders are only partially investigated at the microscopic level, it cannot be excluded that some malignant lesions were missed in this cohort. However, no diagnoses of gallbladder cancer were made during follow-up after cholecystectomy in the current cohort. It cannot be excluded that some of the 148 (5%) patients who did not have a postoperative visit has persistent complaints or complications. However, since these patients never presented back at the hospital, this is unlikely. Finally, the hypothesized reduction in costs by reducing pathology specimens and outpatient visits might not result in a direct cost reduction, but it will likely result in indirect saving of costs. Finally, the macroscopic assessment of gallbladder specimens by the pathologist identified all four gallbladder carcinomas, and whether surgeons are equally effective in identifying macroscopic anomalies remains to be established and might require some training.

In conclusion, pathologic examination of gallbladder specimens appears only useful in case of macroscopic anomalies or in case of a pathology demanding indication for cholecystectomy such as gallbladder polyps or gallbladder mass on preoperative imaging. Although this strategy is included in the national guideline, it has not resulted in the expected reduction in specimens sent for pathology. This change in practice should be included in the informed consent, and the macroscopic examination of the specimen by the surgeon should be documented in the operative report. Also routine follow-up after cholecystectomy appears not useful. Follow-up scheduled only when indicated appears sufficient such as in the case of pathologic examination of the gallbladder specimen or in the case of postoperative complications and could perhaps be performed by the primary care physician. Implementing this strategy will reduce redundant outpatient visits, might decongest the outpatient clinic, and save significant healthcare costs.
